# Pain and Pain Management in Austrian Nursing Home Residents' Daily Lives: A Qualitative Study Guided by an Integrated Quality of Life Model for Older Adults

**DOI:** 10.1111/nhs.70338

**Published:** 2026-04-14

**Authors:** Eva Pichler, Doris Eglseer, Daniela Schoberer, Christa Lohrmann, Sandra Zwakhalen, Manuela Hoedl

**Affiliations:** ^1^ Institute of Nursing and Health Sciences, Medical University of Graz Graz Austria; ^2^ Department of Health Services Research, CAPHRI Care and Public Health Research Institute, Maastricht University Maastricht the Netherlands

**Keywords:** nursing home residents, pain experience, pain management, pain perception, pain self‐management, pain treatment, quality of life

## Abstract

Pain affects various aspects of nursing home residents' lives and their quality of life. The Integrated Conceptual Model of Quality of Life for Older Adults provides a holistic framework to understand factors influencing quality of life. Especially in Austria, knowledge about pain and its management in nursing home residents' daily lives is lacking. Therefore, this study assesses pain and pain management in Austrian nursing home residents' daily lives in the context of the mentioned model. Data were collected in semi‐structured interviews with 28 nursing home residents (median age 85.5/75% female), using an interview guide based on the model. A content‐structuring qualitative content analysis was carried out. Interviewees often report that they did not want to be a burden, indicating that limiting beliefs may contribute to their reluctance to seek help. They emphasized that support from nursing staff was inadequate due to the lack of time, skills, and offered pain management options. Many interviewees used additional interventions to treat pain themselves. Overall, the Austrian situation appears to be consistent with findings in other countries.

## Summary

- Nursing staff must be aware of nursing home residents' attitudes and beliefs about their pain to be able to treat the pain appropriately.
- The behavior of nursing staff influences nursing home residents' pain perception, pain management, and QoL.
- Collaboration between nursing staff and nursing home residents is extremely important for achieving more harmonious alignment in pain management and coordinating pain management efforts.

## 1 Introduction

Nursing homes around the world report pain prevalence rates of up to 85% (Cole et al. 2022; Stompór et al. 2019). Increasing age is often associated with multiple long‐term conditions (e.g., osteoarthritis, spondylosis) and an elevated risk of injury (e.g., due to falls), aspects that increase the likelihood of experiencing pain. Chronic pain, in particular, is more prevalent in people over 65 years than in younger populations (Mills et al. 2019; Mullins et al. 2022). Kumar and Elavarasi (2016) noted that some definitions of the term *pain* are based on a medical description, while others are based on the experience and perception of pain, such as that given by Mccaffery (1968, 95): “Pain is whatever the experiencing person says it is, existing whenever he [she] says it does”.

The perception of pain is influenced by a number of factors, including disease (e.g., depression), as well as by beliefs and attitudes regarding pain (Cole et al. 2022; Schofield et al. 2022). A person in pain often experiences significant suffering, disability, and social isolation; these, in turn, affect their physical and mental capacities, mood, and interest in or ability to participate in leisure activities (Domenichiello and Ramsden 2019; Abeysekera and De Zoysa 2021). Pain, regardless of the type or duration, affects many aspects of life, including physical, psychological or social aspects; therefore, it negatively impacts a person's quality of life (QoL) (Soyuer and Kepenek‐Varol 2019). The Integrated Conceptual Model of Quality of Life for Older Adults (ICMQLOA) divides QoL into six life domains: (1) social, (2) physical, (3) psychological, (4) spiritual, (5) cognitive, and (6) environmental well‐being. The model provides a holistic framework that researchers can use to better understand which factors influence the QoL of older adults. If a negative factor is assigned to one domain, a cross‐domain interaction rather than an isolated impairment will be triggered, thus affecting the entire QoL (Kelley‐Gillespie 2009).

Since pain has a strong (negative) influence on QoL, effective pain management is the key to not only reducing pain but also to improving the QoL of nursing home residents (NHRs) (Helvik et al. 2022). Healthcare professionals providing pain management should focus on an individual's specific pain situation. Interventions should be selected systemically and based on previously gathered information. Among older adults it is recommended to use a combination of non‐pharmacological (e.g., exercises, mind–body interventions, physical interventions) and pharmacological interventions to treat pain in order to avoid excessive or high doses of medication (Levenson et al. 2021). Pain and pain management must be addressed by considering various aspects of life, and not only ways to reduce the pain intensity (Helvik et al. 2022). Pain management interventions initiated by health professionals often address only the physical experience of pain (Holloway et al. 2018) and, while it is important to manage these experiences, other aspects of life that are affected by pain and influence a person's QoL should not be ignored (Wróblewska et al. 2019; Brunkert, Simon, Haslbeck, and Zúñiga 2020). However, pain management interventions initiated by health professionals often address only the physical experience of pain (Holloway et al. 2018).

Services provided in nursing homes vary between countries, but physicians and allied healthcare professionals commonly visit the nursing home rather than work on site (Sanford et al. 2015). The collaboration with healthcare professionals (e.g., physicians) is therefore described by Brunkert, Simon, Ruppen, and Zúñiga (2020) as a barrier to optimal pain management due to the physicians' lack of availability, accessibility, and lack of participation in regular ward rounds. Other barriers include the difficulties nurses face when attempting to assess pain comprehensively in NHRs with communication deficiencies, the lack of training on this subject, and time pressure (Brunkert, Simon, Ruppen, and Zúñiga 2020).

Only some data on pain in nursing homes have been published by public authorities in Austria. These data indicate that pain management in Austrian nursing homes requires improvement. One‐quarter of the assessed nursing homes do not have clear processes for managing or documenting pain, and in every fifth nursing home, no pain assessment scales are used. Furthermore, a lack of training is reported (Ombudsman 2023). The lack of knowledge and false beliefs regarding pain are frequently mentioned problems in Austria (Society 2020). The Austrian Pain Society also indicates that underreporting in the population of older adults in Austria is a major issue. Pain is also often accepted by older adults as a typical and inevitable consequence of aging (Society 2020), and this belief is not limited to Austria (Schofield et al. 2022).

Pain is mostly considered to be a disease, requiring a conventional medical approach rather than human suffering calling for a variety of responses (Ballantyne and Sullivan 2022). Few studies have analyzed pain and pain management as parts of the daily lives of NHRs and embedding them in models that assess QoL such as the ICMQLOA (Kelley‐Gillespie 2009). Healthcare professionals can more clearly understand the consequences of pain and pain management in the NHRs' daily lives and for their QoL, however, if they also understand the role of pain and pain management in the context of the six ICMQLOA domains (Kelley‐Gillespie 2009). This greater understanding may help them recognize all aspects of NHR' pain, choose the most suitable pain interventions, and more effectively meet the individual needs of the NHR regarding the non‐physical aspects of their pain (Haraldstad et al. 2019; Van Leeuwen et al. 2019; Brunkert, Simon, Haslbeck, and Zúñiga 2020). To support this understanding and address the previously mentioned knowledge gap, this study describes pain and pain management in the daily lives of NHRs in Austria in the context for the ICMQLOA as described by Kelley‐Gillespie (2009).

## 2 Materials and Methods

### 2.1 Qualitative Approach

This phenomenological study assumes that pain and QoL are subjective, context‐dependent, and socially constructed experiences. Reality is understood as a concept shaped by individuals' perceptions of pain. Data were collected by asking NHRs to share their pain perceptions and experiences with the aim of representing their subjective realities. The study also adopted a holistic and humanistic view of the persons affected by pain and was theoretically informed by the ICMQLOA as described by Kelley‐Gillespie (2009), which conceptualizes QoL as a multidimensional and person‐centered construct.

Semi‐structured face‐to‐face interviews were performed to better understand the role of pain and pain management in the daily lives of NHRs based on the six ICMQLOA domains described in Kelley‐Gillespie (2009). Interviews are a useful method for recording the nuances of personal interpretations of a topic (Liamputtong 2019), which was the purpose of the survey. The Standards for Reporting Qualitative Research (SRQR) checklist (O'brien et al. 2014) was used for designing and reporting in this study (see Data S1: SRQR Checklist).

### 2.2 Researcher Characteristics

The research team involved in this study consisted of three experienced nursing scientists (M.H., D.E., D.S.), one research assistant, one PhD candidate (E.P.), and one social care worker with additional training in nursing care. One nursing scientist (D.S.) and the PhD candidate are also trained nurses. The nursing scientists and PhD candidate, whose PhD topic is pain in long‐term care, have a special research interest in long‐term care. The researchers are part of a collaborative project between the Medical University of Graz and an Austrian nursing home, and one nursing scientist (M.H.) and the PhD candidate visit this nursing home every week as part of the project. The other nursing scientists (D.E., D.S.) had visited the nursing home several times prior to the beginning of the study; therefore, they were familiar with the setting at the time of data collection. The study took place 8 months after the collaborative project began.

### 2.3 Context

The nursing home where the data collection took place was part of a collaborative project that aims to promote cooperation between research and practice. The nursing home, which has 116 beds in total, is part of a large Christian non‐profit organization located in an urban area of Austria. It is divided into three wards on three different floors. No physicians are on site permanently. Physicians visit their clients either on a regular basis (every other week) or as needed. One physiotherapist works at the nursing home 2 days a week. Nursing care is provided mainly by social care workers (with additional training in nursing care) and healthcare aides. Nurses are mainly responsible for performing administrative tasks; they do not have their office directly on the wards, but instead in a separate wing on the ground floor. At the time of data collection, nurses or other nursing professionals (social care workers or healthcare aides) had not received special pain management training. No structured pain management process had been implemented at this time.

### 2.4 Sampling Strategy

A convenience sample of all NHRs who met the inclusion criteria in one Austrian nursing home were approached directly by the research team and invited to take part in the study. Their participation was voluntary. The inclusion criteria for participating in the study were having experienced pain in the previous 7 days. The research team worked closely together with the nurses and other nursing professionals in the nursing home and discussed their suggestions for participants with them in advance to ensure that those approached would be able to give their written informed consent and/or take part in the study. Furthermore, the nurses and other nursing professionals provided helpful information about the NHRs, that is, that one NHR never wore hearing aids, so the researcher would have to speak loudly.

### 2.5 Data Collection

Face‐to‐face interviews were conducted by two nursing scientists (D.S., D.E.) and a research assistant from 1 February to 31 March 2023. The researchers offered variable interview times, breaks during the interviews, and locations for the interview in the nursing home. The interviewers ensured privacy during the interview, that is, that nobody was within hearing range. The data collection process was carried out based on recommendations provided in the review by Samsi and Manthorpe (2020) for interviewing people living with dementia in social care research. Although this study did not consider persons with dementia, this review includes helpful recommendations and important aspects that should be considered when conducting interviews with people with different levels of cognitive impairment to conduct an ethically sound study. The two nursing scientists involved in collecting the data were trained by the main author (E.P.) to avoid interpersonal differences. All interviews were audio‐recorded, and all NHRs were informed when the recording started or ended. The research team determined when a data saturation point had been reached by reviewing the results after every interview session (Moser and Korstjens 2018). The main author created a list of the main topics mentioned during the interviews in order to identify new topics. Field notes were taken during the interviews to collect information on the interview situation and note special remarks. Optional member checking was offered to the NHRs to ensure the authenticity of the data.

### 2.6 Data Collection Instruments

The main author developed an interview guide on the topic and areas that should be covered for the semi‐structured interviews (see Data S2: Interview guide). This guide is based on the ICMQLO domains and subdomains as described by Kelley‐Gillespie (2009). The model is based on the perspectives of older adults and considers aspects of the nursing home setting in the QoL domains (Kelley‐Gillespie 2009). The first question in the interview guideline asks interviewees to describe their experiences and memories of the last time they have been in pain. One or two main questions about pain were developed for each of the six domains based on the model subdomains. For example, the subdomain named “social relationships/support/contact/interactions/networks/communications” guided the development of the question: “How did you experience support from those around you when you were in pain?” For each question in the guide, additional optional questions are provided based on the subdomains that can be asked to keep the conversation going. Overall, the guide provides eight main questions and 26 optional questions. This guide was pre‐tested with NHRs from the same nursing home, and these NHRs were not later included in the study. The pre‐test results were not included in the current study analysis. Some wording changes were made in the guide to improve understandability based on the feedback received.

### 2.7 Data Processing

Audio records were transcribed by the main author and two independent persons with experience in creating transcripts. The transcription was prepared based on the transcription guideline of Dresing and Pehl (2015) and the semantic‐content transcription system recommended by Kuckartz et al. (2008) was used. Data were anonymized by assigning randomly created codes. Data transfer was carried out according to the Medical University of Graz's internal guideline for data transfer.

### 2.8 Data Analysis

MAXQDA 1989–2021 (MAXQDA, n.d.) was used to conduct the content‐structuring qualitative content analysis based on the recommendations of Kuckartz and Rädiker (2022). The analysis process is shown in Table 1. The coding frame can be found in Data S3: Coding frame.

**TABLE 1:** Steps of the content‐structuring qualitative content analysis based on the recommendations of Kuckartz and Rädiker (2022).

	Step	Description
1	Initiating text work, familiarization with the data	The transcripts were read, irrelevant segments were marked, and if necessary, memos were created.
2	Developing main categories	Thematic categories were developed. The six ICMQLO domains (Kelley‐Gillespie 2009) were used to create the first categories deductively, which were defined as main categories.
3	Data coding with main categories	All transcripts were coded with the main categories.
4	Developing subcategories	All main categories were analyzed individually to further differentiate subcategories inductively. Therefore, lists for each category were generated showing the segments coded with the main categories.
5	Developing the first draft of the coding frame	Main and subcategories were listed in hierarchical order, and then the subcategories were organized systematically. If necessary, some subcategories were summarized into more general subcategories. Definitions were formulated, and examples of the data were added.
6	Data coding with subcategories	All transcripts were coded with the subcategories.
7	Adapting the coding frame	Last changes were made to further improve the coding frame.
8	Check of the coding frame	A second person checked the coding frame by revising the transcripts.
9	Pre‐testing the coding frame	The coding frame was pre‐tested.
10	Finalizing the coding frame	Adaptions were made according to the second person's recommendations and the pre‐test‐results. For example, definitions were modified to clarify the category, or very similar subcategories were summarized.
11	Coding with the final coding frame	The main author coded the transcripts according to the final coding frame.
12	Second coding	The second reviewer coded the transcripts.
13	Category‐based analysis according to the main categories	The leading questions in the step were “What was said on this topic?” and “What was not said on this topic?” The segments of the subcategories were read, checked for similarities or differences and identified specialties.
14	Writing down the results	The first draft of the result section was created.

The quotes referenced in the article were translated into English by a professional translator. The average duration of the interviews was 19 min. In the interviews, the NHRs did not clearly reveal which specific nursing profession (e.g., registered nurses or care aides) was addressed; therefore, the collective term “*nursing staff*” was used to include nurses (e.g., registered nurses) and other nursing professionals (e.g., certified nursing assistants, nursing aides, social care workers). The participants were not involved in the data interpretation process.

### 2.9 Methods Used to Enhance the Rigor of the Interviews

The interview transcripts were analyzed twice by one author (E.P.) within 1 month, and a random sample of transcripts was analyzed by a second reviewer to ensure coding consistency. Discrepancies were discussed. All researchers involved in the study, and especially the main author, spent considerable time in the setting, which enabled them to obtain insights into and a deeper understanding of the setting and interpret the data more effectively. The results were also peer‐debriefed by external researchers. Discrepancies and other alternative interpretations were discussed with these external researchers and the research team.

### 2.10 Ethical Aspects

The study was conducted in accordance with the Declaration of Helsinki and “Good Scientific Practice.” Ethical approval for this study was obtained from the Ethics Committee of the Medical University of Graz (35‐093 ex 22/23). Written informed consent was obtained from each participant or their legal representative before data collection. These individuals were verbally informed by the researchers about the reasons for conducting the study and the researchers themselves. The study included a population of vulnerable people; therefore, a few considerations were made in advance. During the consent process, the researchers asked the NHR questions about the study to ensure that the NHR had understood it. The researchers encouraged them to ask questions at any time and ensured that the NHR had sufficient time to consider whether they wanted to participate. The NHR were informed that participation was voluntary, that their decision had no influence on the care and treatment they received in the nursing home, and that their data would be handled responsibly and anonymously. In Austria, the decision to have a legal representative does not necessarily indicate cognitive impairment; arrangements are often made in anticipation of possible future cognitive decline.

If any questions had arisen regarding a person's capacity to take part in the study during the study period, the process would have been discontinued. The NHRs were informed that they could withdraw from the study without suffering any consequences at any time.

## 3 Results

This section describes results for pain and pain management in the daily lives of Austrian NHRs in the context for the six domains of the ICMQLOA as described by Kelley‐Gillespie (2009).

The majority of the 28 participating NHRs were female (75%); their median age was 85.5 (IQR 81.5–92) years. NHRs mentioned mostly their experiences regarding pain management, namely how nursing staff managed their pain and how they self‐managed it. The categories based on the ICMQLOA (Kelley‐Gillespie 2009) are shown in Table 2.

**TABLE 2:** Categories and subcategories based on the ICMQLO (Kelley‐Gillespie 2009).

Social well‐being	Communication
	Social support
	Time and energy of caregiver
Physical well‐being	Pain and discomfort
	Type of pain management as defined by others
	Side effects of pain management
	Timeliness of care
	Level of physical functioning ability
Psychological well‐being	Coping abilities
	Feelings/emotions/attitude
	Satisfaction with pain management
Spiritual well‐being	Faith/belief in a “higher power”
Cognitive well‐being	Decision‐making ability
Environmental well‐being	Perception of the environment
	Silence, retreat, and privacy

### 3.1 Social Well‐Being

NHRs identified primarily physicians and nursing staff as being part of their pain management, whereby the latter seemed to play a prominent role. However, NHRs reported turning to the nursing staff only when they considered it absolutely necessary. They generally perceived nurses as being under considerable time pressure—especially at night—and as tending to address pain only by administering medication.

No, I do not call. I do not call anyone. No, because they cannot do anything anyway. […] I do not like that; medicines, I do not like taking the pills. No, I just do not like it. I do not like swallowing them. (ID 516)

No, no. I fall asleep and then it stops again. Yes. I cannot say anything. Yes, I mean, how can I say anything? They have enough work to do at night anyway. (ID 474)

NHRs refrained from initiating informal dialogues with the nursing staff regarding their pain, as the nursing staff appeared to be in a state of hurry. In some cases, the participants would discuss their pain with their relatives; however, they attempted to avoid becoming a burden on them.

[…] My son and my daughter are coming sometimes, but I do not want to burden them like that either. (ID 516)

Well, I can [talk about pain with family members], because my children will listen to me. (ID 403)

No. Oh. (…) (sighs) Family. Well, if they ask, ‘How are you?’ Yes. That is just how it is [being in pain]. But I am not going to go on and start complaining about it. They cannot help me anyway. (laughs). (ID 524)

It was further noted that NHRs did not feel able to talk about their pain with other NHRs due to the cognitive impairment of their conversation partners. A number of NHRs specifically mentioned that they did not consider it necessary to discuss their pain.

[…] You do not always HAVE to talk about the pain. You talk about it sometimes, but if you have it all the time, then you cannot keep talking about it. (ID 533)

The majority of the NHRs were aware of the appropriate individuals to contact in order to request pain management. It was emphasized that the paucity of time was a factor contributing to the occasional insufficiency of support from nursing staff. It was reported by several NHRs that the nursing staff were unable to provide assistance, owing to a perceived lack of skills and a restricted range of available pain treatment options.

That does not help me anyway. What do they want to do? They gave me a drink once and a tablet once, and that is all. (ID 595)

Yes, I have it for rubbing on my lower back. On my tailbone. (…) They buy the ointment. They buy it and buy it, but no one rubs it on me. They do not have time. There are too few people here. When you look at how people have to run around all day. I tell you, I could not do that job in this situation. (ID 488)

NHRs who did not mention any negative experiences in the interview regarding their pain management said that they felt as though their requests were taken seriously and that they were understood by nursing staff and relatives. However, the role of family members in supporting individuals in pain management was scarcely addressed.

Yes. Especially at the nursing home, when I had any pain whatsoever, then, yes, they were very concerned about me. Right. So, I can only say the BEST things. (ID 431)

### 3.2 Physical Well‐Being

It was commonly perceived that pain was the cause of various health problems, including those affecting the spine, abdomen, arms, hands, and joints, as well as headaches. A range of less obvious causes of pain was also reported, including pain related to dermatological diseases, shortness of breath, cramps, neurological diseases, or tight clothing/footwear, as well as feelings of tension, numbness, or psychological pain. The majority of NHRs reported experiencing pain during nocturnal hours.

That is at night, when I am asleep […]. I was in so much pain tonight, it is insane. (ID 457)

Yes. It hurts during the day, too, but not as much as at night. At night it is often—how can I put it?—like a seizure. It comes on so quickly. (ID_595)

NHRs asserted that the capability to perform fundamental physical functions, including ambulation and standing, as well as activities of daily living (e.g., writing, dressing, cooking), was either entirely precluded or significantly constrained due to the presence of pain. Furthermore, the disturbance of sleep was cited as a concomitant phenomenon.

[…] I used to hop out of bed and do this and that. And now I can't even get up. (ID 475)

Yes. I mean, cooking or standing for half an hour or whatever … I cannot do that. It is all related to the spine. (ID 474)

I cannot really, let us say, walk and stuff like I used to. Right? (coughs) When I came here in February, when I went for a walk, I went up there, walked all over the mountains. I cannot do that anymore. It is actually gone downhill a lot. Even though I try to get some exercise. Because I have actually always been active my whole life. But I notice that I often cannot manage to walk. So, I just sit down somewhere when I am indoors. There is no other way. (ID 559)

NHRs received pharmacological interventions for pain treatment, sometimes in combination with physiotherapy; however, they rarely described alternative treatments such as massage, natural remedies, cryo‐ or thermotherapy as part of the pain management.

[…] You will get a few tablets here, and that is it. (ID 488)

It appears that NHRs did not consider the adverse effects of the medications to be of significance. Furthermore, no negative associations with the treatment itself were described. Nevertheless, several NHRs expressed a desire for options other than pharmacological ones, typically with the aim of reducing the use of medication.

### 3.3 Psychological Well‐Being

In relation to the management of pain, NHRs outlined a variety of coping strategies, encompassing the application of ointments, the pursuit of increased sleep or rest, the immobilization of the affected body part, or the adoption of a more comfortable position. A range of alternative forms of intervention were also discussed, including physical activity, movement, self‐massage, and distraction. Examples of distraction‐based interventions included television, radio, reading, arts and crafts, and outdoor activities such as gardening.

And I apply a lot of warmth and an ointment. Yes, that helped me. (ID 408)

Then I turn over and sleep it off, so to speak. What I am saying now is nonsense, but that is really how it is. (ID 306)

Well, I have a cushion there anyway, so I prop it [the painful body part] up. (ID 146)

The interviewed NHRs considered their own pain to be negligible and appeared to attempt to stoically endure it for as long as possible. The participants expressed a desire to avoid becoming a burden to others and to maintain their independence. Consequently, they endeavored to self‐manage their pain and refrain from disclosing it to others.

I was always careful not to burden anyone with my pain. So, I always dealt the pain by myself, ALONE. (ID 431)

I suppress it then. I cannot always be asking for things. Because, first of all, it is embarrassing for me, and, second, I do not want to swallow so many medicines. (ID 467)

And I have to accept that (pain). Others have that, too. (ID 488)

Furthermore, during the interviews, some NHRs described expressing pain as “whining” and considered it a habit that should be avoided. The subjects described both their own and others' complaints as unhelpful and a disturbance to others.

Because, I mean, what can she do? She cannot help me now anyway if I complain to her. And all this whining? I just annoy the others with it, too. (ID 474)

What good does it do to complain to others about my pain today? Complaining and whining cannot help. I mean, I know that from others. (ID 474)

The interviewed NHRs reported having had some negative experiences with pain management. In certain instances, the subjects had not received the prescribed treatment, or the treatment had been administered incorrectly. NHRs reported that requests for assistance were, on occasion, disregarded, and that their request for analgesics was forgotten by the nursing staff.

I tell a nurse about it, and she goes out there, and then she does not remember anything about it. When I asked her a day later, because the pain was back again, the nurse said, Jesus, Mary, and Joseph, I forgot! (ID 488)

Yes, I tried. But no one came yesterday/tonight. (…) If the nurse does not come, I am out of luck. (ID 457)

Conversely, NHRs who expressed satisfaction with the support received from nursing staff and their relatives reported that the treatment was beneficial.

Yes, [I've gotten support] in general in the nursing home. When I had any pain, they were very worried about me […]. (ID 431)

After an hour, an hour and a half, it (the pain) is gone. And then, well then the pain is gone for the whole day. (ID 545)

I do not even need to lie down for an hour. If I lie down for half an hour, I feel better again. (ID 559)

### 3.4 Spiritual Well‐Being

A number of NHRs articulated their discontent with regard to their faith in relation to pain. It was frequently asserted that the subjects had endured a considerable number of hardships and, as a result, did not perceive a divine presence to offer solace.

[…] That is just empty talk. I cannot imagine it any other way. And if He were able to help everyone, then we would not all be sick. (ID 474)

No, no. I have lost my faith. Due to my pain and my first husband of the children. Divorced. The second was the good man, but he died. (ID 370)

However, other NHRs also placed their trust in God to alleviate their suffering, employing a combination of prayer and contemplation to seek solace.

I just trust in God and pray for HELP or, when things are bad, I ask Him to help me. (ID 524)

VERY MUCH. That is why I was in the chapel. I prayed to the Virgin Mary. Because I have so much faith in Her. (ID 467)

### 3.5 Cognitive Well‐Being

It was reported by NHRs that they had been given the opportunity to express their personal perspectives on the management of their pain.

Yes, of course. I just say, please, I would like some ointment to be rubbed on. That is, on the shoulder. Of course. (ID 431)

Sure, I could say something. She comes once a week. And all I have to do is to say that I would like to talk to her. Then it is possible. (ID 493)

However, the majority of the NHRs conceded that they were reluctant to assume an active role in the selection of pain management options, citing concerns regarding the adequacy of information provided, their own capacity to make informed decisions, and the involvement of others in the decision‐making process.

I also do not know what I should do. Sometimes it is nerve‐wracking because you do not know anything. (ID 474)

No that is none of my business. I take what I am given. (ID 306)

### 3.6 Environmental Well‐Being

The NHRs did not refer to any negative or positive environmental factors (e.g., light or noise) that influenced their perception of pain.

No, the (noises) do not bother me. (ID 559)

No, I do not know how much the light [bothers me] when it shines in my eyes, but it [the light] does not matter to me. (ID 312)

The NHRs reported that they were able to withdraw from social situations and rest when needed, and that the nursing staff respected their privacy when they were in pain.

I have a place where I have privacy. (ID 488)

No, no one bothers me when I am there. (ID 559)

Oh, quiet. Yes, out there, yes. I sit outside a lot by the HEATER[…]. (ID 408)

Yes, I initially had a twin room. And then I said that, if possible, I would like a single room. Because then I can retreat. When things get really bad, I always lie down in bed. Otherwise, I go outside into nature, into the garden. That helps too. (ID 493)

## 4 Discussion

The aim of this study was to describe pain and pain management in the daily lives of NHRs in Austria, within the context of the ICMQLOA as delineated by Kelley‐Gillespie (2009).

The interviewed NHRs described perceiving pain in a variety of ways and as being caused by a range of health problems. These results again show that pain must be considered as a complex and subjective phenomenon that manifests in a multitude of ways, as has been reported in other studies, for example, by Brunkert, Simon, Haslbeck, and Zúñiga (2020). The study findings suggest that it is critically important for healthcare professionals to recognize and identify the range of underlying causes of pain (Corbett et al. 2016).

The interviewed NHRs appeared to regard their own pain as negligible and as something that had to be endured. The reluctance to request assistance when in pain was attributed to a desire not to impose on others. As Schofield et al. (2022) have demonstrated, older adults frequently hold the belief that they should endure or conceal their pain, which can prevent them from seeking help. The reluctance to impose on others is not only a concern for older adults experiencing pain, but also a more widespread issue. Van Leeuwen et al. (2019) mentioned in their study that older adults are often afraid of being a burden. It was also determined by these authors that, given these adults had become accustomed to independent living and had not previously had to consider another person's time and availability to provide assistance, they frequently encountered difficulties in accepting their need for help (Van Leeuwen et al. 2019). The NHRs interviewed in the present study described the expression of pain as equivalent to “whining,” and considered whining to be something that should be avoided. This indicates the low value that the NHRs placed on their own pain. Schofield et al. (2022) also highlighted the significance of acknowledging an individual's personal experiences with and perceptions of pain, since the attitudes and beliefs of older adults significantly influence their pain management.

The findings of the present study suggest that nurses played a prominent role in the descriptions of the pain management provided by the interviewed NHRs. In cases of pain, NHRs would approach nurses only when deemed absolutely necessary. In this regard, the fact that NHRs perceived and/or experienced that nursing staff lacked time (as well as their experiences with being ignored or forgotten), lacked skills, and could only offer a limited number of pain management options seemed to play an important role in their decision to avoid asking for help or not feeling well‐supported. It is imperative to recognize the significance of social support for older adults, as it has been evidenced to enhance their social well‐being, consequently leading to an improvement in their QoL. Evidence has demonstrated that external support has a positive effect on an individual's ability to cope with challenging circumstances (Kelley‐Gillespie 2009). NHRs value being supported and appreciate being looked after; if this support and appreciation is lacking, this can reduce the QoL (Van Leeuwen et al. 2019; Dagnino and Campos 2022).

The reports of limited numbers of treatment options being offered by the nursing staff and their lack of skills correspond to the findings of Rababa (2018) and Pringle et al. (2021). These authors posited that the decision‐making process in pain management is influenced negatively by a paucity of experience, inadequate training in pain management, or low qualifications. It is evident that these factors increase the level of uncertainty regarding pain management and reduce the ability to confidently make decisions. Consequently, medications are mainly administered for pain (Rababa 2018; Pringle et al. 2021). As previously mentioned in the context section describing the investigated nursing home, nursing care is primally provided by social care workers and healthcare aides, who are professional groups with low qualification levels in healthcare. At the time of data collection, the nursing staff had not received any special training in pain management. Another possible explanation for the results of the current study is cited by Brunkert, Simon, Ruppen, and Zúñiga (2020), namely, that the nursing staff were principally aware of different treatment options, but did not apply more time‐consuming non‐pharmacological interventions due to time constraints. It was frequently observed by the interviewees that the nursing staff appeared to be short of time. This is widely recognized as a significant impediment to the effective management of pain in nursing homes (Brunkert, Simon, Ruppen, and Zúñiga 2020; Rojaye 2024). These factors may provide a rationale for the predominant utilization of pharmacological treatment options, despite the NHRs interviewed in this study employing a wide range of non‐pharmacological self‐management strategies to cope with pain. In this nursing home, it appears that pain was treated by the nursing staff (primarily with medication) and the NHRs themselves (primarily with non‐pharmacological interventions). There is evidence to suggest that there was no collaboration or discussion between the two groups. The NHRs may have sought to address an identified gap in their pain management by initiating their own pain treatment interventions, or they may have aspired to a sense of autonomy in managing the situation. The ability to effectively cope with stressful circumstances and make independent choices are significant factors within the psychological well‐being domain that influence aspects of personal autonomy (Kelley‐Gillespie 2009). Before entering a nursing home, many older adults manage, for example, their medications by themselves. This changes after they move into the nursing home, because the task is performed by the nursing staff (Schwan et al. 2019). This observation may explain why the NHRs in this study could only apply some non‐pharmacological interventions by themselves. Deciding how and when things are done, as well as doing these things independently, contributes to a sense of control that is important for NHRs (Van Leeuwen et al. 2019).

The present study's findings are incongruent with regard to the perceived value of religion or spirituality in the context of pain management. Some NHRs reported experiencing a sense of relief from their pain through religious contemplation, while others indicated that their pain had a detrimental effect on their faith, prompting them to question the existence of a divine being that permits them to endure their state of suffering. This conflict has been reported in another study: Van Leeuwen et al. (2019) posited that faith or spirituality could assist some older adults in accepting their disability and coping with psychological distress or changes in their lives. Other older adults, however, stated that religion was not important to them at all (Van Leeuwen et al. 2019).

The findings of this study offer a novel perspective on the role of pain and its management among older adults living in Austrian nursing homes, thereby contributing to a recognized gap in the literature. While the results are broadly consistent with the existing international evidence, they extend current knowledge by providing contextual insights specific to the Austrian setting. Consequently, the findings add valuable, context‐specific depth to the broader discourse on the role of pain and pain management in nursing home populations.

## 5 Limitations

This study has some limitations. First, the participants were mostly women; thus, the results represent mainly female perspectives, which might differ from male ones. Convenience sampling was used to recruit the NHRs, which is not the preferred approach in qualitative studies (Polit and Beck 2012). These results also cannot be extrapolated to the entire nursing home population in Austria because NHRs with severe cognitive impairment were not included in the sample. Therefore, no inferences can be made regarding the relevance of these findings for this specific group. The risk of interviewer effects in face‐to‐face interviews with older respondents seems to be high according to Kutschar et al. (2022), and these effects might have influenced the quality of the survey data. A response bias may also exist since the answers given in such face‐to‐face interviews are known to change over time. The interview questions asked participants to describe past situations, and the perception of these situations may have changed since the experience was made. Member checks were offered to the participating NHRs, but no responses were received. The NHRs were not included in the data interpretation process; however, the categories were discussed with the social care worker on the research team. The researcher's prior engagement with the setting facilitated a deeper contextual understanding of everyday practices, organizational structures, and interpersonal dynamics. This insider perspective was found to support more nuanced data collection and interpretation, as well as enhance the ability to capture implicit meanings that might otherwise remain inaccessible to external researchers. Concomitantly, we acknowledge the potential risk of bias associated with researcher proximity. In order to address this, reflexive practices were applied throughout the research process, including, for example, the writing of field notes, regular team discussions, and critical reflection with the team and external researchers, in order to ensure both transparency and analytical rigor.

## 6 Conclusion

Austrian NHRs have been found to underreport their pain. Limiting beliefs, such as a reluctance to be a burden or an association between the expression of pain and whining, may contribute to the reluctance of NHRs to seek help. The participants of the study reported that they do not perceive the nursing staff as competent in the management of pain. A review of the international literature reveals that the situation in Austria is consistent with findings in other countries. This demographic constitutes a substantial proportion of the population residing in nursing facilities. The findings of this study also indicate that a stronger focus should be placed on supporting nursing staff in providing pain management to NHRs. For instance, it is necessary to ascertain the competencies required of nursing professionals in order to furnish NHRs with customized and appropriate pain management, as opposed to merely administering pain medication, and to educate them in the provision of pain management to NHRs. In consideration of prospective research endeavors, there is evidently a paucity of knowledge pertaining to the experiences of NHRs afflicted with cognitive impairment.

### 6.1 Relevance for Clinical Practice

It is imperative that Austrian nursing staff possess a comprehensive understanding of the attitudes and beliefs held by NHRs concerning their pain, and that they are able to accurately identify these perspectives. Conversations with NHRs about their previous pain experiences and perceptions may facilitate a more profound understanding of these attitudes among staff. Subsequently, the nursing staff must respond to the identified limiting beliefs and encourage the NHRs to assign a different significance to their pain. The behavior of nursing staff (e.g., lack of time, lack of skills, or few offered treatment options) affects a person's experience of pain and pain management, regardless of whether this behavior is unintentional or intentional. It is therefore essential that nursing staff reflect on their behavior. It is recommended that collaboration between nursing staff and NHRs regarding pain management approaches be encouraged, rather than both parties addressing this issue separately, with a view to achieving more harmonious alignment in pain management and better coordination of pain management efforts. At an organizational level, it is essential to ensure that nursing staff receive adequate training in pain management, enabling them to provide a range of options for treating patients in a variety of pain states. It is submitted that, if the aforementioned points are given due consideration, then adequate attention will be paid to the pain experienced by NHRs.

## Author Contributions

**Eva Pichler:** conceptualization, data curation, formal analysis, investigation, methodology, project administration, visualization, writing – review and editing, writing – original draft. **Doris Eglseer:** investigation, funding acquisition. **Daniela Schoberer:** funding acquisition, investigation. **Christa Lohrmann:** supervision, visualization, writing – review and editing. **Sandra Zwakhalen:** writing – review and editing, visualization, supervision. **Manuela Hoedl:** data curation, funding acquisition, investigation, methodology, project administration, supervision.

## Funding

This work is supported by the Austrian Science Fund (FWF), Vienna, Austria as part of the #ConnectingMinds program (Grant CM 300‐G). Funders provided financial support for this forthcoming research but are not and will not be involved in the conducting or how the research is conducted.

## Ethics Statement

The study was approved be the Ethic Committee of the Medical University of Graz (EC Number 35‐093ex22/23).

## Consent

All participating nursing home residents, or their legal representative, had to give written consent bevor data collection.

## Conflicts of Interest

The authors declare no conflicts of interest.

## Supporting information


**Data S1:** The Standards for Reporting Qualitative Research (SRQR) checklist (O'brien et al. 2014). This checklist was used for designing and reporting the study.

**Data S2:** The interview guide used for the interviews with the nursing home residents. The guide is based on the Integrated Conceptual Model of Quality of Life for Older Adults described by Kelley‐Gillespie (2009). For each of the six domains, questions were developed. Overall, the guide provides eight main questions and 26 optional questions.

**Data S3:** The coding frame for the content‐structuring qualitative content analysis based on the recommendations of Kuckartz and Rädiker (2022).

## Data Availability

The interview data used to support the findings of this study have not been made available because this was stipulated in the sponsorship agreement.
