# Supplementary material for: Pain and Pain Management in Austrian Nursing Home Residents' Daily Lives: A Qualitative Study Guided by an Integrated Quality of Life Model for Older Adults
Source: Nurs Health Sci. 2026 Apr 14;28(2):e70338. doi: 10.1111/nhs.70338 (PMC13080236; doi:10.1111/nhs.70338)
Supplement: Supplementary file 2 — Data S2: The interview guide used for the interviews with the nursing home residents. The guide is based on the Integrated Conceptual Model of Quality of Life for Older Adults described by Kelley‐Gillespie (2009). For each of the six domains, questions were developed. Overall, the guide provides eight main questions and 26 optional questions. [file NHS-28-e70338-s001.docx]

**Supplementary Material B: Interview guide**

**Key question/stimuli/prompt:** As already mentioned, I am interested in how pain affects your life and your everyday life. We are under no time pressure, so you can talk freely and I may ask a few questions in between if necessary. To begin with, I would like you to think back to a situation in which you experienced pain. Please tell me how you experienced the pain and what you can remember.

| **Content aspects according to the domains of the conceptual model of quality of life of Kelley-Gillespie (2009)** | **Question** | **Special maintenance questions** | **General maintenance questions** |
| --- | --- | --- | --- |
| **Social aspects** | How did you experience support from those around you when you were in pain? | How did you experience the support from your carer?  How did you experience the support from your family/friends?  Did you feel understood?  Did you feel that you were taken seriously?  Were you able to talk to someone about your pain? If so, with whom?  Did you know who you could turn to for pain treatment? If so, who did you turn to? | Can you tell me more about that?  How exactly did you experience it? |
| **Physical aspects** | What was done for your pain?  Did the pain treatment and care you received help you? | What kind of treatment did you receive?  Was anyone else consulted to alleviate your pain?  Did you feel that the pain treatment and care were satisfactory for you?  Did you receive the treatment promptly?  Did you have less pain after the treatment?  Did you feel any restrictions or side effects from pain medication? |  |
| **Psychological aspects** | To what extent can you take measures to relieve the pain yourself? | To what extent were you able to control how you dealt with the pain yourself?  What specific pain-relieving measures were you able to take yourself?  Can you explain these in more detail? |  |
| **Spiritual aspects** | Does your faith or spirituality help you when you are in pain? | Are there any religious or spiritual rituals that you perform when you are in pain? |  |
| **Cognitive aspects** | Were you able to contribute to the choice of your pain therapy? | How did you experience how the nursing staff dealt with your decisions regarding pain treatment?  Were you able to choose between several treatment options?  Were you able to make your own suggestions as to which type of treatment would help you? Were these respected? |  |
| **Environmental aspects** | How did you perceive your surroundings when you were in pain?  Were you able to retreat to a comfortable place when you were in pain? | Think of sounds, or noises, how did you perceive them?  Was there anything disturbing/pleasant about it?  Was there anything in your environment that did you good when you were in pain?  Was there anything in your environment that affected you negatively when you were in pain?  What opportunities did you have to withdraw in order to have privacy or rest?  Was your wish for privacy/rest respected?  By whom was your wish respected/not respected? |  |
| **Open questions** | Are there any other aspects that influence your quality of life? | Are there other things that do or do not do you good in your everyday life? |  |
| **End** | Ask if the person would like to add anything else. Ask if there is a question for the interviewer. Thank them and inform them what will happen next. If necessary, accompany the person back to the room/living area. | | |

KELLEY-GILLESPIE, N. 2009. An integrated conceptual model of quality of life for older adults based on a synthesis of the literature. *Applied Research in Quality of life,* 4**,** 259-282
