# Supplementary material for: Pain and Pain Management in Austrian Nursing Home Residents' Daily Lives: A Qualitative Study Guided by an Integrated Quality of Life Model for Older Adults
Source: Nurs Health Sci. 2026 Apr 14;28(2):e70338. doi: 10.1111/nhs.70338 (PMC13080236; doi:10.1111/nhs.70338)
Supplement: Supplementary file 3 — Data S3: The coding frame for the content‐structuring qualitative content analysis based on the recommendations of Kuckartz and Rädiker (2022). [file NHS-28-e70338-s002.docx]

**Supplementary Material C: Coding frame**

|  | **Category** | **Definition according to Kelley-Gillespie (2009)** |
| --- | --- | --- |
| Category | Social well-being | Includes sociocultural status, political environment, financial status factors, standard of living, material possessions/resources/supports/circumstance, possession or attainment of tangible things, social relationships/support/contact/interactions/networks/communications, daily activities/recreation/leisure, opportunities for fun, humor, enjoyment, and creativity, age appropriate activities, continuity of past with continuation of social roles, sense of connectedness between home, neighborhood, and community, contact with statutory/voluntary organizations, community interactions, time and energy of caregiver, commitment/flexibility of caregiver, the way care is given, interaction/relationship between caregiver and care recipient, compatibility, balance of power, boundary maintenance, Inside–out/outside–in activities, sense of belonging. |
| Sub category | Communication | If a nursing home resident wants or does not want to talk to somebody about pain or pain management. As well as asking for help or treatment. |
| Sub category | Social support | If a nursing home resident gets support from others or not, as well as the nursing home resident does not want any support from others. Furthermore, this sub category contains information about how seriously the nursing home resident feels being taken and all persons, involved in pain management and offering support. |
| Sub category | Time and energy of caregiver | Quality of the relationship between the caregiver and the care recipient is an important indicator of social well-being, which is often affected by the length of the relationship and the time and energy put in by the caregiver. This category includes if the nursing home resident gets the time and energy from the caregivers, which is needed to treat their pain. |
| Category | Physical well-being | Includes physical well-being may encompass physical health, personal hygiene, nutrition, exercise, grooming, clothing, general appearance, physical condition and functioning level of an individual, pain and discomfort, level of caregiving and caregiving interventions, diagnosis, prognosis, symptoms, medication and side effects. |
| Sub category | Pain and discomfort | This category summariness all information a nursing home resident is giving regarding his/her pain. |
| Sub-sub-categories | Situation of pain | When does the pain occur? |
|  | Frequency of pain | Nursing home resident’s description of how often they are in pain. |
|  | Type of pain | Nursing home resident’s pain description and reason for pain. This could be whatever the resident says it is. |
|  | Pain intensity | Nursing home resident’s pain intensity description. |
| Sub category | Type of pain management set by others | What intervention are set to treat pain by health professionals, friends or relatives for pain relief regarding the nursing home resident. |
| Sub category | Side effects of pain management | Nursing home resident’s annotation regarding unpleasant effect, noticed when getting pain treatment, as well as they do not have any unpleasant effects (side effects due to pharmacological or non-pharmacological treatment). |
| Sub category | Timeliness of care | This category includes information regarding the time frame within a nursing home resident receives pain management. |
| Sub category | Level of physical functioning ability | The effect of pain on the persons’ ability to perform; affected activities of daily living by pain. |
| Category | Psychological well-being | Includes emotional and mental health, feelings, evaluation concerning the self, self-esteem, self-concept, constitutes psychological well-being, vitality and capacity to identify internal and external resources in the face of stressful situations, tangible support, affection, positive interaction, informational support, emotional support, personal autonomy, subjective satisfaction, personality, life satisfaction/level of life acceptance, happiness, extent to which goals were achieved, and level of satisfaction with care, support, programs, and services. |
| Sub category | Coping abilities | This category summarizes all coping abilities a nursing home resident are able to set by themselves. |
| Sub category | Feelings/emotions/attitude | Nursing home resident’s feelings/emotions/attitude regarding pain. |
| Sub category | Satisfaction with pain management | Nursing home resident’s view on the things going well and not going well at the pain management set by themselves and others. Nursing home resident’s feeling about the efficacy of pain management set by themselves and others. |
| Category | Spiritual well-being | Includes affirmation of life in relationship with God, self, community and environment, one’s personal values or morals, standards of conduct and spiritual belief, senses of connectedness and faith or belief in a “higher power”. |
| Sub category | Faith/ belief in “higher power | Faith/ belief/ spirituality/ religion as part of pain management, that helps or helps not a nursing home resident when in pain to feel relief. |
| Category | Cognitive well-being | Includes the thinking process and management skills of the mind, intellectual capacity and ability to make decisions and judgements. Includes the standard of housing or institutional living arrangements, control over physical environment, access to facilities (shops, public transportation), safety, secure, usable, accommodation, comfortable, clean, affordable, level of privacy, level of personalization and hominess. |
| Sub category | Decision making | This category includes information regarding the ability and possibility for a nursing home resident to get involved in the pain management. |
| Category | Environmental well-being | Includes the standard of housing or institutional living arrangements, control over physical environment, access to facilities (shops, public transportation), safety, secure, usable, accommodation, comfortable, clean, affordable, level of privacy, level of personalization and hominess. |
| Sub category | Perception of the environment | Nursing home resident’s perception of the environment when he/she is in pain, as well as any preferred changes in the environment to feel better when in pain. |
| Sub category | Silence, retreat and privacy | Nursing home resident’s possibility to get silence or privacy when he/she is in pain and having the possibility to retreat. |

KELLEY-GILLESPIE, N. 2009. An integrated conceptual model of quality of life for older adults based on a synthesis of the literature. *Applied Research in Quality of life,* 4**,** 259-282.
